# Navigating the Deep Eutectic Solvent Landscape: Experimental and Machine Learning Solubility Explorations of Syringic, p-Coumaric, and Caffeic Acids

**DOI:** 10.3390/ijms262010099

**Published:** 2025-10-16

**Authors:** Piotr Cysewski, Tomasz Jeliński, Maciej Przybyłek, Natalia Gliniewicz, Marcel Majkowski, Michał Wąs

**Affiliations:** Department of Physical Chemistry, Pharmacy Faculty, Collegium Medicum of Bydgoszcz, Nicolaus Copernicus University in Toruń, Kurpińskiego 5, 85-096 Bydgoszcz, Poland; piotr.cysewski@cm.umk.pl (P.C.); m.przybylek@cm.umk.pl (M.P.); 310370@stud.umk.pl (N.G.); 310396@stud.umk.pl (M.M.); 310431@stud.umk.pl (M.W.)

**Keywords:** deep eutectic solvents, phenolic acids, solubility prediction, machine learning, nuSVR, COSMO-RS, molecular descriptors, model complexity, AICc

## Abstract

Efficiently identifying suitable solvents for active pharmaceutical ingredients (APIs) is critical in drug formulation, yet the vast number of possible solvent-solute combinations presents a significant experimental challenge. This study addresses this by developing a robust machine learning (ML) model for accurately predicting the solubility of three phenolic acids (syringic, p-coumaric, and caffeic) in various deep eutectic solvents (DESs), integrating both experimental and computational investigations. Measured solubility data showed that the choline chloride combined with triethylene glycol in a 1:2 molar ratio was the most efficient system for the dissolution of the studied APIs. Different ML models, utilizing nu-Support Vector Regression (nuSVR) as the core regressor and based on descriptor sets derived from COSMO-RS (Conductor-like Screening Model for Real Solvents) computations, were systematically evaluated. A novel methodology termed DOO-IT (Dual-Objective Optimization with ITerative feature pruning) was employed to address the common challenges of model development with limited, high-value datasets. The final optimal 10-descriptor nuSVR model, selected from an exhaustive, multi-run search, demonstrated outstanding predictive power, offering a highly reliable computational tool for guiding experimental screening, significantly accelerating the exploration of DES-based formulations. This research also provides a strong foundation for future machine learning-guided discovery of chemicals, offering an effective and transferable framework for developing QSPR models for various chemical systems.

## 1. Introduction

Phenolic acids are a prominent group of naturally occurring plant secondary metabolites with a wide range of biological activities and industrial applications [1,2,3]. Among them, caffeic acid, syringic acid, and p-coumaric acid are three structurally related hydroxycinnamic and hydroxybenzoic acid derivatives extensively studied for their antioxidant, antimicrobial, anti-inflammatory, and anticancer properties. These compounds are ubiquitous in fruits, vegetables, grains, and beverages, including coffee, wine, and tea, and have garnered attention in recent years for their potential therapeutic and nutraceutical uses [4,5]. Caffeic acid (3,4-dihydroxycinnamic acid) belongs to the hydroxycinnamic acid class and is characterized by a catechol moiety and a propenoic acid side chain. This structure results in high radical scavenging activity and metal-chelating ability, which underlie its potent antioxidant effects [6,7]. Caffeic acid occurs both in its free form and as an ester with quinic acid, most notably in the form of chlorogenic acid in coffee beans. It exhibits anti-inflammatory effects through inhibition of cyclooxygenase enzymes and the NF-κB signaling pathway, with additional effects on glucose metabolism modulation and protection against oxidative stress-related diseases [8,9,10]. Syringic acid (4-hydroxy-3,5-dimethoxybenzoic acid) is a derivative of benzoic acid and belongs to the hydroxybenzoic acid subclass. Its structure includes two methoxy groups on the aromatic ring, which influence its antioxidant potential and lipophilicity. Syringic acid is commonly found in red wine, olives, as well as acai berries and has been reported to exhibit antioxidant, anti-inflammatory, neuroprotective, and antimicrobial activities [11,12,13]. Its neuroprotective effects have been linked to the reduction of reactive oxygen species (ROS) and inhibition of apoptosis in neuronal cells, making it a promising compound for neurodegenerative disease research [14,15]. p-Coumaric acid (4-hydroxycinnamic acid) differs structurally from caffeic acid by having only one hydroxyl group on the aromatic ring. It is widely distributed in cereal grains, tomatoes, or honey and acts as a precursor in the biosynthesis of more complex phenolics such as lignin and flavonoids [16,17]. Its biological properties include antioxidant, anti-inflammatory, and antimicrobial effects, and it has been found to inhibit lipid peroxidation and modulate the enzyme activity involved in detoxification and inflammatory pathways [18,19]. Recent studies have also explored its role in enhancing food preservation and packaging due to its ability to inhibit microbial growth and oxidative spoilage [20]. Together, these three phenolic acids share a common biosynthetic origin via the phenylpropanoid pathway [21,22] and exhibit structural similarities that contribute to overlapping, yet distinct, biological profiles.

**Figure 1:**
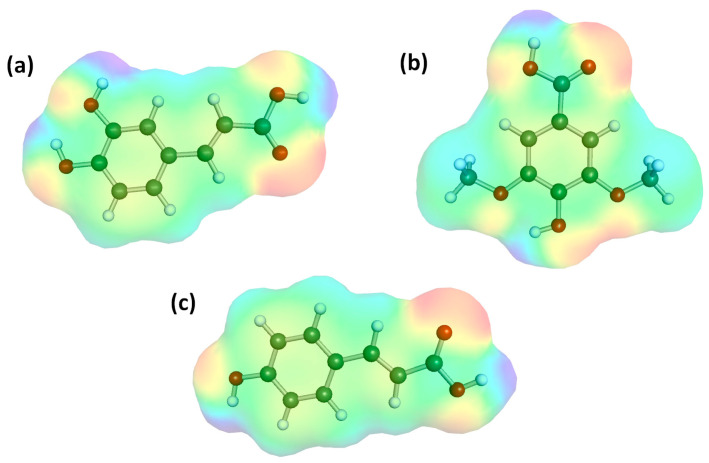
Molecular electrostatic potential (MEP) maps of (**a**) caffeic acid, (**b**) syringic acid, and (**c**) p-coumaric acid, illustrating the distribution of electron density across the molecular surface. The color scale represents the electrostatic potential: red areas indicate regions of negative potential, blue areas correspond to positive potential, and green areas represent regions of near-neutral potential.

The solubility behavior of caffeic, p-coumaric, and syringic acids is primarily governed by their distinct substitution patterns on the aromatic ring, which influence polarity, hydrogen-bonding capacity, and hydrophilicity. The structural formulas and electron density distributions of the three considered phenolic acids are presented in Figure 1. Caffeic acid is a phenylpropanoid with an ortho-dihydroxy moiety and a conjugated propenoic acid chain. The two adjacent hydroxyl groups act as strong hydrogen bond donors and acceptors, enabling extensive interaction with polar solvents such as water, alcohols, and aqueous-organic mixtures. Consequently, caffeic acid generally exhibits the highest solubility in hydrophilic environments but tends to have limited solubility in apolar media due to strong self-association via intermolecular hydrogen bonding. p-Coumaric acid contains a single hydroxyl substituent in the para position, resulting in a reduced capacity for hydrogen bonding and a moderately lower polarity compared with caffeic acid. This structural simplification decreases its aqueous solubility but slightly improves compatibility with less polar solvents. Syringic acid, in turn, features one hydroxyl and two methoxy groups on the aromatic ring. The methoxy substituents introduce steric bulk and increase lipophilicity while reducing the number of available hydrogen-bond donors. As a result, syringic acid is typically less soluble in highly polar solvents such as water (although its structure makes it more soluble than caffeic acid), but its solubility may improve in moderately polar or protic organic media where dipole–dipole or π–π interactions can stabilize the solute. The combination of hydroxyl substitution, methoxy functionalization, and overall structure of these compounds explains the general solubility behavior of the three acids. It also accounts for their varying, although limited, aqueous solubility. Syringic acid can be characterized by the highest solubility in water, due to its more compact benzoic structure and the presence of methoxy groups, followed by caffeic acid and p-coumaric acid.

Given their pharmacological potential and broad natural occurrence, these phenolic acids have attracted increasing interest in pharmaceutical research. However, their limited water solubility mentioned earlier remains a major challenge for practical applications, including formulation development. In this context, deep eutectic solvents (DESs), particularly natural deep eutectic solvents (NADESs), have been identified as promising media for enhancing the solubility of phenolic acids and related bioactive compounds. Deep eutectic solvents are a class of solvents typically composed of two or more compounds, with at least one of them acting as a hydrogen bond acceptor (HBA) and the other as a hydrogen bond donor (HBD), which, when mixed at a specific molar ratio, form a liquid mixture with a melting point significantly lower than those of its individual components [23,24]. In this work, we follow the widely accepted convention in green chemistry and solvent technology by using the term “deep eutectic solvents” (as well as the abbreviation DESs) to refer to all binary or ternary mixtures composed of HBAs and HBDs, regardless of their specific molar ratio. While only the formulation that exhibits the lowest possible melting temperature strictly qualifies as the eutectic mixture from a purely thermodynamic standpoint, the term DES is widely employed as a convenient and established descriptor for this entire class of low-melting, biodegradable solvents.

These solvent systems significantly enhance the solubility of poorly water-soluble compounds through specific interactions such as hydrogen bonding and dipole–dipole interactions between solvent and solute molecules [25,26]. DESs also improve the stability of phenolic compounds during storage and digestion, which protects them from degradation and preserves their antioxidant and bioactive properties [27,28,29]. Furthermore, the use of NADESs as extraction and formulation media has been shown to markedly increase the bioavailability of phenolic constituents, with reported improvements of up to 140% in the case of anthocyanins extracted from blueberries compared to conventional methods [27]. The biocompatibility, biodegradability, and safety profile of DESs support their suitability for use in pharmaceutical, food, and cosmetic applications, reinforcing their value in the development of functional foods and nutraceutical formulations [25,30,31,32,33]. There are many specific instances of DES utilization across multiple industrial sectors. In cosmetics, companies such as Gattefossé have reported development and commercial use of DES-based extraction technologies to produce natural botanical actives, and Naturex has commercialized DES-extracted botanical collections for personal care. Pharmaceutical companies, such as Abbott Laboratories, Nuvo Research Inc., or Lipocine Inc., utilize DESs to create more effective drug delivery systems, particularly for poorly soluble drugs. Bioeutectics, on the other hand, is a company that produces DESs from bio-derived raw materials and is positioned as a supplier of green solvents for consumer packaged goods (CPG) in the cosmetic, food, and pharmaceutical industries. These examples underscore that DESs are not merely academic curiosities but are entering the market as components of end-use products.

Deep eutectic solvents, and particularly natural deep eutectic solvents, made from biobased HBAs and HBDs, are frequently proposed as greener alternatives to volatile organic solvents and some ionic liquids because they can be prepared from inexpensive, readily available components and often exhibit low vapor pressure [24,34]. Compared with conventional organic solvents such as ethanol, acetone, hexane, or toluene, DESs typically display negligible volatility and flammability, which minimizes risks associated with emissions, explosion hazards, and operator exposure [35]. Moreover, the use of natural hydrogen-bond donors and acceptors (e.g., sugars, amino acids, organic acids, or choline derivatives) enables the design of biodegradable and potentially food-grade solvent systems, unlike many petrochemical solvents that are often toxic and subject to strict regulatory limits [30]. However, when considering DESs as an alternative to classic solvents, a balanced perspective is necessary. First, toxicity and biodegradability are composition-dependent: some DES formulations (e.g., those based on carboxylic acids) show ecotoxicity and require careful safety evaluation, whereas sugar- or polyol-based NADES tend to be less harmful [36,37]. Second, while raw material costs of typical DES components are low and the preparation is simple, process costs associated with separation, solvent recovery, and mitigation of high viscosity (e.g., addition of co-solvents, heating, membrane separation) can negate economic advantages upon scale-up [38,39]. Third, mass transfer limitations due to high viscosity remain significant engineering challenges and are addressed through back-extraction strategies, membrane processes, and choice of hydrophobic DES variants [40,41]. Finally, regulatory acceptance for food and pharmaceutical applications is not yet universal, and each DES-based formulation requires individual toxicological and regulatory assessment prior to product approval. Overall, DESs offer promising environmental and formulation advantages, but their adoption in industry requires holistic evaluation of safety, processing and recovery costs, as well as regulatory pathways.

To support rational design and application of DESs, predictive modeling has become an area of active research. Machine learning enables accurate prediction of key physicochemical properties of DESs, such as viscosity, heat capacity, density, and solubilizing capacity, across a wide range of compositions and conditions, offering both high precision and broad applicability [42,43,44,45,46,47,48,49,50,51,52]. An effective strategy for predicting solubility in DESs involves integrating such techniques with the COSMO-RS (Conductor-like Screening Model for Real Solvents) approach, which enables the calculation of molecular interactions based on quantum chemical descriptors. This approach uses molecular interaction data from COSMO-RS, such as σ-profiles and intermolecular interaction energies, as input features for machine learning models including random forests, support vector regressors, and neural networks [44,48,49,50,51,53,54].

The current study addresses the issue of limited solubility of three phenolic acids (syringic, p-coumaric, and caffeic) by means of experimental and computational investigations. Experimental efforts were focused on identifying the optimal deep eutectic solvent composition for phenolic acid dissolution, which involved exploring both neat DESs and their aqueous mixtures. Computational studies were aimed at developing an effective and robust machine learning model, which would accurately predict solubility in various DES formulations. These investigations, although focused on particular systems, can serve as a foundation for future research and the development of a more general predictive solubility model based on machine learning techniques.

## 2. Results and Discussion

### 2.1. Experimental Solubility

During the first phase of experiments, the solubility of three considered phenolic acids was studied in neat deep eutectic solvents at 25 °C. Owing to the usage of different hydrogen bond acceptors (HBAs) and donors (HBDs) and their various molar proportions, twenty-four distinct DES systems were investigated for each acid, allowing the identification of clear general patterns among the obtained results. When taking into account the influence of the HBA, assuming the same HBD and molar ratio, choline chloride (ChCl) outperformed betaine (Bet). On the other hand, when considering the effectiveness of various HBDs, independently of the used HBA and molar ratio, triethylene glycol (TEG) turned out to be the most efficient, while ethylene glycol (ETG) was the least effective, with glycerol (GLY) and diethylene glycol (DEG) in between. The only exception to this pattern was the case of p-coumaric acid in betaine-based DES systems, in which GLY was responsible for the highest solubility. Finally, the 1:2 HBA:HBD molar ratio gave the highest solubility of the studied phenolic acids, with the 1:4 molar ratio being the second most effective and the 1:1 molar ratio yielding the lowest solubility, provided that DES constituents remain the same. When looking at detailed solubility values, expressed as the mole fraction of a particular compound, the p-coumaric (COU) acid was characterized by the highest solubility, amounting to x_COU_ = 0.0997 with a standard deviation (SD) value of 0.0009 in the most effective choline chloride-based system, i.e., ChCl-TEG 1:2, and x_COU_ = 0.0898 (SD = 0.0003) in the optimal betaine-based eutectic, i.e., Bet-GLY 1:2. Caffeic acid (CAF) was slightly less soluble with x_CAF_ = 0.0835 (SD = 0.0010) for ChCl-TEG 1:2 and x_CAF_ = 0.0755 (SD = 0.0010) for Bet-TEG 1:2 and syringic acid (SYR) had the lowest solubility with x_SYR_ = 0.0515 (SD = 0.0004) for ChCl-TEG 1:2 and x_SYR_ = 0.0474 (SD = 0.0003) for Bet-TEG 1:2. Detailed solubility values can be found in Supplementary Materials (Tables S1.1–S1.3).

The second phase of measurements was devoted to the investigation of phenolic acids’ solubility in aqueous DES systems. For this task, two best-performing eutectic systems, one with choline chloride and one with betaine, were selected and mixed with water in various proportions (expressed by the mole fraction of the DES in the mixture ranging from x*_DES_ = 0.1 to x*_DES_ = 0.9). The experimental temperature was in the range from 25 °C to 40 °C with 5 °C intervals. Again, some general patterns in the behavior of the studied systems were observed. First of all, rather unsurprisingly, the increase in temperature yielded higher solubility of the three phenolic acids. When taking into account the neat DES (x*_DES_ = 1.0) in 25 °C and 40 °C, the solubility increase for p-coumaric acid was 244% for the DES with choline chloride and 205% for the system involving betaine. In the case of caffeic acid, this increase was 231% and 161%, respectively, while for syringic acid, the corresponding values were 240% and 164%. More importantly, the amount of water in the eutectic system also influenced the solubility. In general, adding water to a DES system results in a decrease in the solubility of the active pharmaceutical ingredient. However, a small amount of water added to the eutectic can often increase solubility. This behavior was observed by our research team for other systems [49,55,56], and this was also the case for the phenolic acids studied here. The origins of these observations are not obvious, but they can be related to specific intermolecular interactions within the systems, particularly those between choline chloride and water. This phenomenon is not yet widely studied in the literature [57,58] and deserves a separate investigation, which we are aiming to perform in the future. In this particular study, in the case of each studied eutectic system and all considered phenolic acids, the aqueous DES in which the amount of the eutectic corresponded to a composition of x*_DES_ = 0.9 (i.e., when the water content was x_water_ = 0.1) was the most effective solubilizer, although the solubility increase, compared to neat DES, was not very pronounced. In the case of p-coumaric acid, the solubility in the ChCl-TEG 1:2 system at this optimal composition was found to be x_COU_ = 0.1054 (SD = 0.0008), which corresponds to a 6% increase in the solubility compared to the neat DES, while for the Bet-GLY 1:2 system, the solubility value was x_COU_ = 0.0926 (SD = 0.0006), amounting to a 3% increase in the solubility compared to the pure eutectic. For caffeic acid, the corresponding solubility values were x_CAF_ = 0.0851 (SD = 0.0005, 2%) and x_CAF_ = 0.0823 (SD = 0.0013, 9%), while for the syringic acid, this was x_SYR_ = 0.0550 (SD = 0.0005, 7%) and x_SYR_ = 0.0488 (SD = 0.0004, 3%). In higher temperatures the solubility increases were similar. Detailed solubility values can be found in Supplementary Materials (Tables S1.4–S1.6).

It is also important to analyze the above results in the context of solubility values found in the literature. The solubility of syringic [59,60,61,62], p-coumaric [63,64,65,66], and caffeic [62,63,66,67,68,69] acids was studied in various solvent systems, including water, organic solvents, and ionic liquids. In the case of p-coumaric acid, the solubility values achieved in this study were higher not only than those in water but also than those found in the most effective organic solvent, i.e., methanol. Only some imidazolium-based ionic liquids achieved higher or comparable solubility, although the usage of such ionic liquids is debatable due to their potential toxicity [70]. Again, for caffeic acid, the eutectic systems outperformed water and all classical organic solvents (including the most efficient acetone), with only the bmimBF_4_ ionic liquid achieving greater solubility. Similarly, in the case of syringic acid, the studied DESs were found to be more effective solubilizers than water and all organic solvents. This comparison with available literature data highlights the effectiveness of deep eutectic solvents as alternative solubilizing media for various pharmaceutically active compounds.

### 2.2. Model Performance and Evaluation

**Figure 2:**
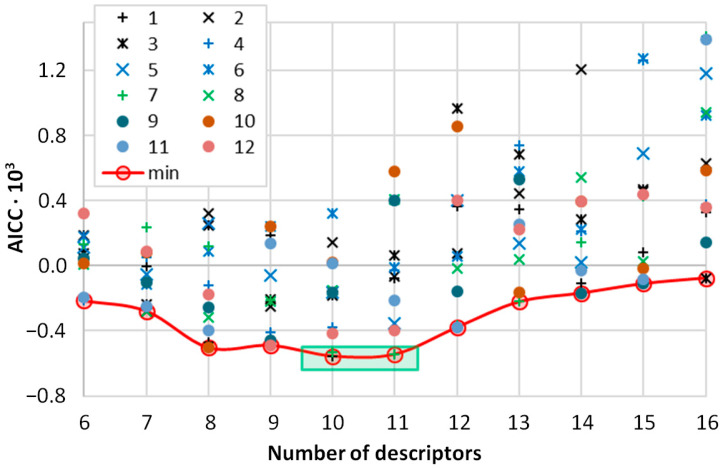
Stability analysis of the DOO-IT model selection procedure. The plot displays the corrected Akaike Information Criterion (AICc) for the optimal model found at each stage of iterative feature pruning across twelve independent runs. Each symbol represents a unique model selected from a Pareto front. The red line traces the envelope of the best-performing models (lowest AICc) found at each level of descriptor complexity. The clear convex shape of this envelope reveals a “basin of excellence” (highlighted by the green box) where the most parsimonious and accurate models are consistently located, demonstrating the robustness of the methodology in identifying an optimal model with 10 descriptors.

The study utilized a solubility dataset (N = 1148) characterizing saturated systems of the studied phenolic acids across various DES formulations, neat solvents, and binary solvent mixtures. In addition to the DES solubility values measured specifically for this study, the values obtained earlier for ferulic acid [48] were included, as well as solubility data available in the literature for these phenolic acids [59,60,61,62,63,64,65,66,67,68,69,71,72,73,74]. This dataset defines the applicability domain of this work, which was not aimed at formulating a universal solubility model but rather at constructing a robust, validated, and highly predictive QSPR model specifically tailored for the screening of novel deep eutectic solvents for a defined class of chemical compounds, namely phenolic acids. The computational methodology was therefore designed to create a tool that can reliably navigate this specific chemical space, accelerating the discovery of optimal DES formulations for this important class of bioactive compounds, rather than attempting to extrapolate to unrelated systems where the underlying physicochemical interactions may differ significantly. Indeed, the developed systematic Dual-Objective Optimization with ITerative feature pruning (DOO-IT) procedure is immune to both the target property and the set of descriptive features. Here, DOO-IT was used to find machine learning models simultaneously fulfilling two requirements: accuracy and transferability. The results of this extensive model search are presented in Figure 2.

Models were constructed using nu-Support Vector Regression (nuSVR), a choice driven by its proven efficacy in handling non-linear relationships and its robustness against overfitting, which is crucial for small to medium-sized datasets, making it particularly suitable for the prediction tasks in this study. As it was documented in the methodology section, the final decision of model selection was made using the corrected Akaike Information Criterion (AICc). The intention of selecting this criterion, rather than using a simple number of support vectors, was to define a more general and model-agnostic criterion. However, the regressor-specific complexity is used for pre-selection of model candidates. The robustness and credibility of our final models are the direct result of the DOO-IT methodological design, which rests on three synergistic pillars. Each pillar addresses a critical challenge in developing trustworthy QSPR models from limited, high-value datasets.

The first pillar is the Dual-Objective Optimization. Instead of optimizing for predictive accuracy alone, which invariably leads to overly complex models, simultaneous optimization was conducted for both accuracy (minimizing the Mean Absolute Error (MAE)) and model simplicity (minimizing a complexity metric, such as the support vector ratio to the size of the training data). This approach does not produce a single “best” model, but rather a Pareto front of equally optimal solutions, forcing a direct and transparent confrontation with the bias-variance trade-off from the outset.

The second pillar is the iterative feature pruning. Recognizing that the optimal hyperparameters are dependent on the feature set, our procedure does not perform feature selection as a single, preliminary step. Instead, it iteratively prunes the single least informative feature, identified via permutation importance, and then re-runs the entire dual-objective optimization on the reduced feature set. This ensures that the features and hyperparameters are co-adapted, leading to a much more rigorous and holistic search for the most parsimonious and powerful model.

The third and final pillar is the information-based model selection. From the large family of candidate models generated across all iterations, the ultimate best model is selected based on a fundamental statistical principle, namely the corrected Akaike Information Criterion. By analyzing the AICc trajectory as a function of model complexity (as shown in Figure 2), we can objectively identify the point at which the loss of predictive power from removing a feature outweighs the benefit of increased simplicity. This allows for a final, data-driven, and model-agnostic decision that pinpoints the single model with the most justifiable balance of accuracy and parsimony.

The results of our twelve independent DOO-IT runs are summarized in Figure 2, which plots the AICc for the model selected at each stage of feature pruning. The wide scatter of outcomes for any given number of descriptors immediately reveals the challenging, non-convex nature of simultaneous feature and hyperparameter optimization. This variance underscores that different pruning pathways and hyperparameter combinations can lead to vastly different models, highlighting the risk of relying on a single optimization run. Despite this complex and non-convex landscape, a clear and powerful trend emerges. The envelope of the best-performing models, indicated by the red line, forms a distinct convex curve. This line represents the best possible model found at each level of complexity and provides decisive evidence for an optimal trade-off: models that are too simple (6–9 features) or too complex (12+ features) are consistently and demonstrably inferior. The curve does not exhibit a sharp minimum but rather a broad region of optimal model complexity, highlighted by the green box, which spans from 10 to 11 features. This demonstrates a critical finding: while the performance of any single run exhibits significant variance, the globally optimal solutions discovered by the DOO-IT procedure are exclusively located within this parsimonious region. The methodology, therefore, serves as a powerful framework for proving that for this system, the most stable and accurate models are also among the simplest. This comprehensive mapping of the solution landscape provides an exceptionally robust justification for our final model selection.

**Figure 3:**
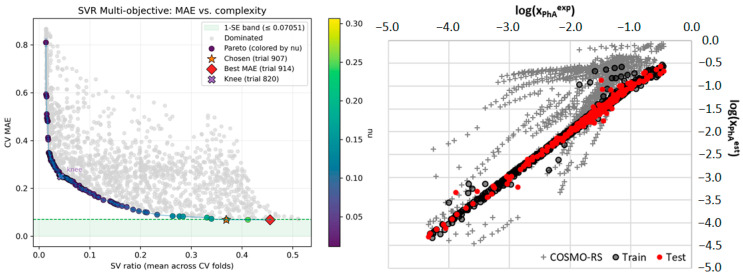
Final model selection and performance validation. (**Left panel**) The dual-objective optimization results for the run that produced the final, selected model. Each point represents a model’s cross-validated Mean Absolute Error (MAE) versus its complexity (support vector ratio). The Pareto front (dark purple) represents the optimal trade-off solutions. The final model (trial 907, orange star) was selected using the 1-Standard Error (1-SE) rule, identifying it as the most parsimonious model within the 1-SE band (green shaded region) of the most accurate model (trial 914, red diamond). (**Right panel**) Parity plot comparing the model’s predicted (est) versus experimental (exp) solubility (log mole fraction, x) for the training set (black circles) and the held-out test set (red circles). The high correlation and tight clustering around the line of perfect agreement (y = x) demonstrate the model’s excellent predictive power and generalization. Predictions from the baseline COSMO-RS model are included for comparison (grey crosses).

Based on this comprehensive stability analysis, the single model exhibiting the globally minimum corrected Akaike Information Criterion was selected for final validation and implementation. This optimal model, which utilizes 10 descriptors, represents the most statistically robust and parsimonious solution discovered across the twelve independent DOO-IT runs. In Figure 3 there are presented the details of this particular model. The left panel illustrates the outcome of the dual-objective optimization for the specific run from which the final model was selected.

Each point represents a unique nuSVR model, plotted according to its cross-validated MAE and its complexity. The dark purple points form the Pareto front, representing the set of models with the best possible trade-off between accuracy and simplicity. All other models (grey) are considered suboptimal. The green shaded region indicates the 1-Standard Error (1-SE) band, calculated from the model with the lowest MAE on the Pareto front (trial 914). Our final model selection adheres to the 1-SE rule, which favors parsimony by selecting the simplest model whose performance is statistically indistinguishable from the most accurate model. In this case, trial 907 was chosen as the optimal model, as it is the least complex solution within this band. This rigorous selection process ensures that the chosen model is not only highly accurate but also maximally parsimonious, a key prerequisite for robust generalization. On the right panel of Figure 3, the parity plot is presented for the final selected model (trial 907), comparing its predicted solubility values against experimental data for both the training (black circles) and the held-out test sets (red circles). The data points are tightly clustered around the line of perfect agreement across a wide range of over four orders of magnitude, demonstrating the model’s high predictive power and lack of systematic bias. The performance on the unseen test set is visually indistinguishable from the training set, providing strong qualitative evidence of the model’s excellent generalization capabilities. Furthermore, the plot clearly shows the substantial improvement of our nuSVR model over the baseline COSMO-RS predictions provided as grey crosses, which exhibit significantly higher scatter and systematic deviations, particularly at the extremes of the solubility range. A detailed error analysis reveals that the few noticeable outliers correspond to specific, challenging physicochemical systems. For instance, in the lowest solubility region (log(x) < −4.0), the model slightly overestimates the solubility of syringic acid in water, a system where strong, directional hydrogen bonding and entropic effects are notoriously difficult to capture. Conversely, in the highest solubility region (log(x) > −0.5), the model shows minor deviations for caffeic acid in DES comprising choline chloride with acetic acid or 1,4-butanediol. These systems represent the boundaries of the model’s applicability domain, where the underlying interactions may be uniquely complex. The model’s excellent performance on the vast majority of systems, coupled with an understanding of these specific outliers, reinforces its utility as a reliable screening tool for typical DES formulations. The details regarding the performance of the optimal model can be found in Section S3 of the Supplementary Materials.

To provide a comprehensive and rigorous assessment of the final 10-descriptor model, a two-pronged validation strategy was employed. The results of this analysis, presented in Table 1, demonstrate the model’s exceptional robustness. First, the model’s stability and expected generalization performance were quantified using a 5-fold cross-validation procedure on the entire training dataset. The cross-validated R^2^ was 0.976 ± 0.004, with the low standard deviation indicating that the model’s high performance is not sensitive to the specific subset of data used for training. The model also achieved a very low cross-validated Mean Absolute Error (MAE) of 0.072 ± 0.004. Second, the single, final model trained on all available training data was evaluated on the held-out, completely unseen test set. On this final exam, the model achieved an R^2^ of 0.984 and a MAE of 0.061. The Root Mean Square Error (RMSE) of the model was found to be 0.116 ± 0.007 for the cross validation and 0.125 for the held-out test set.

The excellent agreement between the cross-validated performance and the final test set performance provides the ultimate confirmation of the model’s validity. It proves that the model has learned a statistically significant and generalizable relationship, and its high predictive power is not an artifact of a single data split but a true reflection of its capabilities.

**Figure 4:**
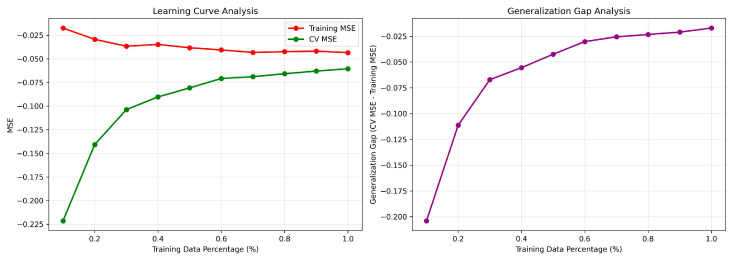
Learning Curve Analysis for the optimal nuSVR model. (**left panel**) presents the mean squared error (MSE) as a function of training set size. (**right panel**) shows the generalization gap representing the absolute difference between training and test scores at each subset size.

Additionally, the generalization capabilities of the model are presented in Figure 4 in the form of a learning curve analysis for the optimal nuSVR model, providing definitive evidence of its robust generalization capability and the success of the model validation protocol. The learning curves exhibit a nearly ideal profile for a well-generalized machine learning model. As mandated by the validation protocol, the Mean Squared Error (MSE) for the training and cross-validation sets converges decisively to a stable, asymptotic value as the size of the training subset increases (left panel). The most critical metric, i.e., the generalization gap (right panel), is acceptably small and narrows consistently with more data, demonstrating that the model has learned the underlying physical principles governing solubility in DESs rather than memorizing noise or idiosyncrasies in the training data.

In addition to predictive accuracy, the selected model offers significant practical advantages. The inclusion of COSMO-RS-computed solubility as a descriptor improves the accuracy of the model, highlighting the value of physically meaningful descriptors. This supports the broader applicability of such descriptors in other solubility systems. Despite the inherent limitations of COSMO-RS computations, especially their dependence on accurate fusion data and computational cost, their integration in the nuSVR model transforms them from weak standalone predictors into important high-value descriptors. This modeling approach enables reliable extrapolation to unmeasured systems, making it suitable for use in computational pre-screening workflows that reduce the experimental burden. These results underline that even when the direct solubility values computed from COSMO-RS are imperfect, their informational content remains significant. Overall, the synergy between experimental data and well-engineered descriptors processed via machine learning proves to be a robust strategy for predicting complex physicochemical properties such as solubility.

Nonetheless, despite the significant advantages offered by the COSMO-RS computed solubility, it is imperative to acknowledge inherent limitations associated with its computation. A critical prerequisite for accurate solubility prediction using COSMO-RS is the availability of reliable fusion data. Unfortunately, these data are frequently unavailable, as they have not yet been measured. Particularly for novel, less-studied compounds or those synthesized in small amounts, these values are inaccessible. Additionally, there are cases in which measured values suffer from strong discrepancies. This, in turn, leads to uncertainty in the necessary values of fusion Gibbs free energies. There are even more fundamental restrictions in cases where such data are simply unavailable or ill-defined. For example, this issue arises in the case of different polymorphs existing at the melting point and under ambient conditions or for stable solvates under saturated solution conditions but unstable at melting conditions. In such scenarios, the “melting point” of the active pharmaceutical ingredient as a pure solid is not the relevant solid form in equilibrium with the saturated solution, making fusion data for the pure API inapplicable or non-existent in a simple sense. These limitations highlight a practical bottleneck for broad-scale, purely computational screening, emphasizing the need for robust strategies when such essential input data are missing or ambiguous.

## 3. Materials and Methods

### 3.1. Materials

The three phenolic acids used in the study, namely caffeic acid (CAF, CAS: 331-39-5, MW = 180.16 g/mol, ≥98% purity), syringic acid (SYR, CAS: 530-57-4, MW = 198.17 g/mol, ≥95% purity), and p-coumaric acid (COU, CAS: 501-98-4, MW = 164.14 g/mol, ≥98% purity) were supplied by Sigma Aldrich (Saint Louis, MO, USA). The deep eutectic solvents (DESs), which were used for the dissolution of the above active pharmaceutical ingredients, were composed of a hydrogen bond acceptor (HBA) and a hydrogen bond donor (HBD) in different molar ratios. The utilized HBAs included choline chloride (ChCl, CAS: 67-48-1, ≥99%) and betaine (Bet, CAS: 107-43-7, ≥98), both obtained from Sigma Aldrich. Four different HBDs were used, namely ethylene glycol (ETG, CAS: 107-21-1), diethylene glycol (DEG, CAS: 111-46-6), triethylene glycol (TEG, CAS: 112-27-6), and glycerol (GLY, CAS: 56-81-5), which were also supplied by Sigma Aldrich with a purity of ≥99%. Methanol (CAS: 67-56-1, analytical grade) was used as a secondary solvent and was delivered by Chempur (Piekary Śląskie, Poland). All the chemicals were used without any additional procedures.

### 3.2. Experimental Solubility Measurements

In order to determine the solubility of the three studied phenolic acids in deep eutectic solvents, a very well-established shake-flask method coupled with spectrophotometric measurements was utilized.

The initial procedure involved the preparation of various DESs, which was conducted by mixing their HBA and HBD counterparts in a glass vessel and heating the composition until the formation of a transparent, uniform liquid. Two HBAs (ChCl and Bet) mixed with four HBDs (ETG, DEG, TEG, GLY) in three molar ratios (1:1, 1:2, 1:4) resulted in a total of 24 different eutectic systems. For additional measurements, the DESs were mixed with water in varying proportions, creating aqueous DES systems. Saturated solutions of CAF, SYR, and COU were obtained by adding excess amounts of these compounds to test tubes containing either a neat deep eutectic solvent or its aqueous solution. Such samples were placed in the Orbital Shaker Incubator ES-20/60 from Biosan (Riga, Latvia) and heated for 24 h at 25 °C, 30 °C, 35 °C, and 40 °C (±0.1 °C) with simultaneous mixing at 60 rpm. Before measurements, the samples were filtered using a PTFE syringe filter from Qpore (Dallas, TX, USA) with a 0.22 µm pore size. In order to avoid possible precipitation of the saturated solutions at decreased temperatures, the test tubes, syringes, pipette tips, and filters were kept at a temperature identical to that of the samples.

The spectrophotometric measurements required the preparation of calibration curves for the three APIs. This involved the preparation of a stock solution of each compound in methanol, which was later successfully diluted, and the obtained solutions of varying concentrations were measured using an A360 spectrophotometer from AOE Instruments (Shanghai, China). The wavelength range was set to 200–500 nm, and the resolution was 1 nm. The analytical wavelengths were determined to be 324 nm for caffeic acid, 273 nm for syringic acid, and 308 nm for p-coumaric acid. The final regression equations, obtained after averaging three distinct sets of measurements, are collected in Table 2 together with determination coefficients and limits of detection (LOD) and quantification (LOQ) for the calibration curves.

The measurements of the samples were conducted with the same spectrophotometer and identical settings. Methanol was used for the dilution of the samples so that they could remain within the linearity range of the method. Complete spectra of the samples were recorded, but for the solubility determination, the absorbance values at a specific analytical wavelength were considered. These values were combined with the corresponding regression equations of the calibration curves and served for the calculation of the concentration of the three phenolic acids in the studied samples. The densities of such samples were also measured, and for this task 1 mL of each sample was weighed to a precision of 0.1 mg using an AS 110 R2.PLUS analytical balance from RADWAG (Radom, Poland). The obtained densities enabled the calculation of mole fractions of CAF, SYR, and COU dissolved in the studied DESs. For each considered system, three distinct measurements were conducted, and the results were averaged.

### 3.3. Molecular Descriptors

Training of non-linear models requires appropriate representation of the molecular structure. The selection of molecular descriptors is a critical task, necessitating adherence to several general requirements: (i) descriptors must be derivable solely from molecular structure, without the need for experimental data; (ii) they must incorporate temperature dependence; (iii) they ought to account for mixture compositions; and (iv) they should possess clear physical meaning. Requirement (i) is particularly crucial as it enables the screening of virtually any compound or solvent, including those not yet synthesized or experimentally characterized. Adhering to these restrictions also allows for adaptability to various experimental conditions and aids in the interpretation of solute-solvent interactions, thereby enhancing the understanding of factors determining equilibrium. All these requirements are met by COSMO-RS-derived properties, as demonstrated in our previous studies [48,49,51]. Herein, molecular descriptor values were derived using COSMOtherm version 24.0.0 [75] from solubility computations within the COSMO-RS framework [76,77,78,79]. The typically applied protocol utilizes an iterative procedure and is supposed to be fast and reasonable. Unfortunately, in the case of deep eutectic solvent systems, where mole fractions of APIs in saturated systems are often very high and comparable to the amount of the solvent, this method fails. Indeed, for highly soluble solutes, complete miscibility [80,81,82,83] is incorrectly predicted. Hence, to prevent such inconsistencies, a complete solution of the solid–liquid equilibrium (SLE) has been applied to all systems under investigation. This significantly increases computation time, often by at least an order of magnitude, posing a challenge for large datasets but is necessary for obtaining a consistent descriptor dataset. Besides, successful computation of solubility in COSMO-RS requires providing fusion data for each solid solute considered. Hence, the values of the Gibbs free energy of fusion are to be included in the input files. The values were derived based on melting temperature, T_m_, and heat of fusion, ΔH_fus_, which were collected from those available in the literature [84]. In the case of multiple data, the averaged values were used. The heat capacity of fusion was assumed to be constant and was calculated as follows: ΔCp,fus ≈ ΔSfus ≈ ΔHfus/Tm. For reproducibility purposes, all values of Gibbs free energy of fusion, ΔGfus = ΔHfus − TΔSfus, used for solubility computations were collected in the Supplementary Materials (please refer to the SM-dataset spreadsheet). Hence, the first choice descriptors were the computed values of solubility. Besides, from the last iteration data in the output files, energetic descriptors were extracted, including the total intermolecular interactions as well as contributions from electrostatic (misfit), hydrogen bonding (HB), and van der Waals (vdW) forces. Also, the values of chemical potentials (μ) were included. Thus, five descriptors were used for the solute: E_int,API_, E_misfit,API_, E_HB,API_, E_vdW,API_, and μ_API_. Similar data were used for the characterization of solvents, defining descriptors as the sum of each DES component weighted by its molar fraction in the solute-free solution. Finally, the relative values, defined as the difference between solute and DES descriptors, were also included. Hence, in total, 16 descriptors were used for model training purposes.

The values of molecular descriptors were computed for all DES containing the studied phenolic acids (N = 543), which comprised those collected from literature and augmented with results measured for the purpose of this study. Additionally, for increasing the solvent space, the solubilities of these phenolic acids in neat solvents (N = 318) and binary solvent mixtures (N = 287) were included. Hence, the dataset used for model development consisted of N = 1148 data points. All data, including solubility values and molecular descriptors, are available in the SM_dataset spreadsheet and are explained in the Section S2 of the Supplementary Materials.

### 3.4. Machine Learning Protocol

#### 3.4.1. Model Development Framework

The development and evaluation of the models followed a rigorous computational protocol designed to ensure a robust performance and reliable generalization. Firstly, for the regression task, nu-Support Vector Regression (nuSVR) [85] was selected as the core machine learning regressor among many available algorithms. This choice was motivated by its proven efficacy in handling non-linear relationships and high-dimensional data, common characteristics of chemical property prediction problems. Although nuSVR incorporates the “kernel trick” [86], allowing it to model complex, non-linear relationships by mapping data into higher-dimensional spaces, here only a Radial Basis Function (RBF) kernel was used as the regressor. The RBF kernel was chosen due to its proven effectiveness in a wide range of QSPR problems, its ability to capture complex non-linearities, and its established success as a default, high-performing kernel in cheminformatics. It implicitly maps samples into an infinite-dimensional feature space, giving it the flexibility to model highly complex decision boundaries with a single, well-behaved hyperparameter. The hyperparameters optimized during this process were C (regularization parameter) and nu. The gamma hyperparameter (RBF kernel coefficient), which controls the influence of individual training samples, was optimized using a guided, data-driven approach rather than a direct, unconstrained search. At the beginning of each iteration, a heuristic gamma_base value was calculated based on the median pairwise squared Euclidean distance between a subset of points in the current training data [87,88,89]. The optimizer then searched for an optimal logarithmic scaling factor (log10_gamma_scale) in the range [−1.5, 1.0] to refine this data-driven anchor. This two-step strategy ensures that the search for gamma is focused on a physically meaningful and appropriately scaled range, significantly improving the efficiency and effectiveness of the optimization.

Secondly, data standardization was performed on all molecular descriptors [90]. As Support Vector Machines (SVMs), including nuSVR, are not scale-invariant, it is crucial to scale the input features to prevent features with larger absolute values from disproportionately influencing the model. Each feature was standardized by removing the mean and scaling to unit variance, a process implemented using StandardScaler from the scikit-learn library. This ensures that all descriptors contribute equally to the distance calculations within the kernel space. Thirdly, the prepared dataset (N = 1148 data points) was divided into training and testing sets. A conventional 80% of the data was allocated for training the models, while the remaining 20% was reserved as an independent test set for final, unbiased evaluation of the selected models’ performance on unseen data. To ensure reproducibility of this split, a fixed random_state was utilized.

#### 3.4.2. Dual-Objective Optimization (DOO): Accuracy vs. Complexity

The core of the model development is a Dual-Objective Optimization (DOO) strategy, designed to systematically direct the trade-off between predictive accuracy and model simplicity. The Optuna framework version 3.2 [91,92] is employed for this task, utilizing the Tree-structured Parzen Estimator (TPE) sampler. For each candidate model, two objective functions were simultaneously minimized. The first one targeted the predictive error, which was the Mean Absolute Error (MAE) calculated via 5-fold cross-validation on the training dataset. MAE was chosen for its robustness to outliers and its direct interpretability in the units of the target variable. The second objective addressed the model complexity. This is a regressor-related quantity, and here the mean support vector (SV) ratio across the 5 folds was chosen. The SV ratio for a single fold is defined as the number of support vectors in the trained model divided by the total number of samples in that fold’s training set. For a nuSVR, the SV ratio is a direct, intrinsic measure of model complexity, representing the fraction of training data points required to define the regression function. A lower SV ratio indicates a simpler, less complex model.

#### 3.4.3. Iterative Feature Pruning and Candidate Selection

A key innovation of the developed framework is the integration of feature selection directly into the optimization loop. Instead of a single feature selection step, we employed an iterative backward pruning strategy. The procedure begins with the full set of molecular descriptors. After a complete dual-objective optimization is performed, the single best model on the resulting Pareto front is identified. This selection is made using the 1-Standard Error (1-SE) rule, a statistically robust heuristic for model selection [90,93]. First, the model with the lowest MAE on the Pareto front is identified, tentatively representing the most accurate model. The 1-SE threshold is then defined as the best MAE and its associated standard error. The chosen model for that iteration is the one with the lowest complexity (minimum SV ratio) whose MAE falls within this threshold. This process favors parsimony by selecting the simplest model that is statistically indistinguishable from the most accurate one. Following the selection of this model, its features are ranked using permutation importance [94] with 10 repeats on the training set. The single feature with the lowest importance score is pruned, and the entire dual-objective optimization is repeated on the reduced feature set. This iterative cycle continues until a predefined minimum number of features is reached, generating a candidate model at each stage of complexity.

#### 3.4.4. Information-Based Model Selection

The iterative pruning procedure generates a family of high-performing candidate models, one for each level of feature complexity. To make the final, objective selection, a model-agnostic, information-theoretic criterion was employed, namely the Akaike Information Criterion (AIC) [95], and specifically its small-sample corrected variant, AICc [96].

These information criteria provide a principled framework for assessing the relative quality of statistical models for a given set of data, balancing model fit against the number of estimated parameters to mitigate the risk of overfitting. The fundamental principle behind AIC is to estimate the information lost when a candidate model is used to represent the process that generated the data; lower AIC/AICc values indicate less information loss and, thus, a relatively superior model within the candidate set. The standard formulations for AIC and AICc are defined as:

(1) $$\mathrm{AIC}=2k-2ln\left(\hat{L}\right)$$

(2) $$\mathrm{AICc}=\mathrm{AIC}+\frac{2k(k+1)}{n-k-1}$$

where *k* represents the number of parameters in the model, $ln\left(\hat{L}\right)$, is the natural logarithm of the maximum likelihood estimate for the model given the data, and *n* is the number of data points (observations). AICc introduces an additional penalty term for model complexity, which is particularly important when the sample size (*n*) is small relative to the number of parameters (*k*), typically when *n*/*k* < 40. In such scenarios, AICc provides a more accurate and reliable assessment of model quality. It is important to acknowledge that nuSVR models, unlike traditional statistical models, do not arise from a direct probabilistic framework with an explicit maximum likelihood function. Therefore, a pseudo-Akaike Information Criterion corrected (pseudo-AICc) approach was adopted for their evaluation, building upon established heuristics for non-probabilistic machine learning models. For the purpose of calculating the pseudo-AICc, the natural logarithm of the maximum likelihood ($ln\left(\hat{L}\right)$) was approximated by assuming a Gaussian error distribution for the model’s residuals. Under this assumption, $ln\left(\hat{L}\right)$ can be estimated from the Residual Sum of Squares (RSS) and the number of observations (*n*) as follows:

(3) $$\mathrm{AIC}=2k-2ln\left(\hat{L}\right)\approx n\cdot ln\left(\sigma^{2}\right)+n\left(\frac{\mathrm{RSS}}{s\cdot \sigma^{2}}-1\right)+2k$$

where $\sigma^{2}\;$= RSS/*n* is the estimated error variance. The most critical aspect for SVM-based models is the definition of the effective number of parameters, *k*. In nuSVR, the model’s complexity is primarily determined by the number of support vectors, which are the training data points that directly influence the position and orientation of the regression function. Therefore, for the pseudo-AICc calculation, *k* was defined as the total count of support vectors identified by the fitted nuSVR model, augmented by two additional parameters: one for the bias (intercept) term inherent in the regression function, and another for the estimated error variance ($\sigma^{2}$). This heuristic provides a tangible measure of model complexity that incorporates both the core structural elements of nuSVR and the necessary statistical components for a likelihood approximation. This pseudo-AICc quantification allowed for a robust, quantitative comparison of nuSVR models trained with varying descriptor sets, providing a critical metric for model selection in this study.

A pseudo-AICc value was calculated for each candidate model, and by plotting the AICc for the best model found at each complexity level, one can identify the model that optimally balances fit and parsimony. The model with the globally minimum AICc value across all runs and iterations was selected as the final model for subsequent validation and analysis. This final selection step ensures that our model is not only accurate and simple but also statistically justified.

All calculations and analyses were performed using Python version 3.10 [97] and key libraries including scikit-learn [91], Optuna [92], and pandas [98]. The DOO-IT procedure was implemented as a fully automated framework. To ensure an exhaustive search of the solution space, the stability analysis was conducted by performing twelve independent repetitions of the entire DOO-IT procedure. Within each of these repetitions, every dual-objective optimization step (one for each feature pruning iteration) was run for 2000 trials, leading to a comprehensive collection of candidate models for the final AICc-based selection.

## 4. Conclusions

The current study is devoted to the problem of limited solubility of three phenolic acids (syringic, p-coumaric, and caffeic), particularly to the efforts aimed at efficient identification of suitable solvent candidates for improved dissolution of these compounds. This work addresses the issue by developing a robust machine learning model for accurately predicting the solubility of the considered phenolic acids in various deep eutectic solvents (DESs), integrating both experimental investigations and computational insight. The ability to accurately predict solubility for phenolic acids within DES, even for previously uncharacterized combinations, provides a powerful computational guide for exploring the vast experimental space, significantly reducing the need for exhaustive and resource-intensive laboratory trials.

The initial experimental investigations provided a comprehensive evaluation of the solubility behavior of the studied APIs in a wide range of DES systems. The results clearly demonstrated that solubility is significantly influenced by the nature of both hydrogen bond acceptors and donors, as well as their molar ratios. Among the studied HBAs, choline chloride was generally more effective than betaine when paired with the same donor, while in terms of HBDs, triethylene glycol exhibited the highest solubilizing capacity in most cases. The optimal HBA:HBD molar ratio was found to be 1:2, consistently yielding the highest solubility across all systems, while the 1:1 ratio was the least effective. Additionally, the incorporation of small amounts of water into the eutectic systems (corresponding to an aqueous DES composition at x*_DES_ = 0.9) resulted in slightly enhanced solubility, further improving the performance of the already optimized neat DESs. A comparison with literature data revealed that the developed DES systems offered superior solubility for all three phenolic acids relative to water and traditional organic solvents, including the most effective ones, such as methanol and acetone. Only certain imidazolium-based ionic liquids matched or exceeded the solubility levels observed in DESs, however, their practical use can be limited due to toxicity concerns. These findings underscore the high potential of deep eutectic solvents as green, efficient, and tunable solubilizing media for poorly water-soluble bioactive compounds. The identified trends and optimal formulations offer valuable insights for further experimental and computational exploration of DES-based pharmaceutical applications.

The key result of the study was the successful development and rigorous validation of a high-performance QSPR model for predicting the solubility of phenolic acids in a diverse range of deep eutectic solvents and other solvent systems. The final 10-descriptor nuSVR machine learning model, selected from an exhaustive, multi-run search, demonstrated outstanding predictive power, achieving a cross-validated R^2^ of 0.976 ± 0.004 and a final R^2^ of 0.984 on a held-out, unseen test set. This result represents a significant step forward in the in-silico screening and rational design of sustainable solvent formulations for this important class of bioactive compounds. Additionally, this rigorous framework unequivocally demonstrated that the integration of the COSMO-RS computed solubility, despite its standalone inaccuracy, acts as a transformative, high-information descriptor. Within our nuSVR model, it serves as a physicochemical anchor, guiding the algorithm to achieve superior accuracy with reduced complexity.

The success of this endeavor is underpinned by a novel, systematic methodology we termed DOO-IT (Dual-Objective Optimization with ITerative feature pruning). This framework was specifically designed to address the common challenges of model development with limited, high-value datasets. By synergistically combining Dual-Objective Optimization (accuracy vs. simplicity), Iterative feature pruning, and Information-based model selection relying on the corrected Akaike Information Criterion (AICc), the DOO-IT procedure provides a robust and objective pathway to discovering maximally parsimonious and generalizable models. Our comprehensive stability analysis, performed over twelve independent runs, confirmed that despite a complex optimization landscape, this methodology consistently identifies an optimal region of model complexity, providing an unprecedented level of confidence in the final selected model.

While the predictive model itself is expertly tailored to its applicability domain of phenolic acids, the DOO-IT framework is a universal and transferable contribution. It serves as a general-purpose recipe for building trustworthy machine learning models in chemistry, materials science, and other fields where data is precious and the risk of overfitting is high. The speed and robustness of the developed workflow now open the door for rapid exploration of new chemical spaces. Future work will focus on applying this powerful methodology to other challenging solute-solvent systems and integrating it into broader computational workflows for accelerated materials discovery.

Finally, it is essential to contextualize the applicability domain of the developed model. The final 10-descriptor model is a highly specialized tool, expertly trained on the physicochemical space defined by phenolic acids within choline chloride- and betaine-based deep eutectic solvents. Its high predictive accuracy is therefore expected to be maintained for solutes and solvents that are structurally and chemically similar to those in the training set. Consequently, applying the model to vastly different chemical classes, such as non-acidic APIs or entirely new types of solvents, would constitute an extrapolation beyond its validated domain and should be approached with caution. This clear definition of the model’s boundaries is not a limitation but a feature, ensuring its responsible and effective use as a targeted screening tool. The DOO-IT framework, however, provides a clear and robust blueprint for systematically expanding this domain as new, diverse experimental data becomes available, defining a clear path for future work in developing a more universal solubility model.

## Figures and Tables

**Table 1:** Final performance metrics of the selected optimal 10-descriptor nuSVR model.

Metric	5-Fold Cross-Validation (on Training Set)	Held-Out Test Set (Final Evaluation)
R^2^	0.976 ± 0.004	0.984
MAE	0.072 ± 0.004	0.061
RMSE	0.116 ± 0.007	0.125

**Table 2:** Details of the calibration curves used for solubility measurements.

Phenolic Acid	Regression Equation (C in [mg/mL])	R^2^	LOD [mg/mL]	LOQ [mg/mL]
caffeic	A = 62.51 × C − 0.0126	0.9993	0.00072	0.00215
syringic	A = 111.64 × C − 0.0094	0.9987	0.00046	0.00138
p-coumaric	A = 54.27 × C − 0.0106	0.9983	0.00139	0.00417

## Data Availability

The original contributions presented in this study are included in the article/Supplementary Materials. Further inquiries can be directed to the corresponding author.

## Supplementary Materials

The following supporting information can be downloaded at: https://www.mdpi.com/article/10.3390/ijms262010099/s1.
