# Supplementary material for: Navigating the Deep Eutectic Solvent Landscape: Experimental and Machine Learning Solubility Explorations of Syringic, p-Coumaric, and Caffeic Acids
Source: Int J Mol Sci. 2025 Oct 16;26(20):10099. doi: 10.3390/ijms262010099 (PMC12564265; doi:10.3390/ijms262010099)
Supplement: Supplementary file 1 [file ijms-26-10099-s001.zip › ijms-3916992-supplementary/supplementary_materials.pdf]

Supplementary Materials

Navigating the Deep Eutectic Solvent Landscape: Experimental and Machine Learning Solubility Explorations of Syringic, p-Coumaric, and Caffeic Acids

Piotr Cysewski, Tomasz Jeliński*, Maciej Przybyłek, Natalia Gliniewicz, Marcel Majkowski, and Michał Wąs

S1. Solubility measurements 2

Table S1.1. Mole fraction solubility of coumaric acid (COU) in deep eutectic solvents composed of choline chloride or betaine as the hydrogen bond acceptor (HBA) and one of four polyols as the hydrogen bond donor (HBD) at different molar ratios, measured at 25 °C. 2

Table S1.2. Mole fraction solubility of caffeic acid (CAF) in deep eutectic solvents composed of choline chloride or betaine as the hydrogen bond acceptor (HBA) and one of four polyols as the hydrogen bond donor (HBD) at different molar ratios, measured at 25 °C. 2

Table S1.3. Mole fraction solubility of syryngic acid (SYR) in deep eutectic solvents composed of choline chloride or betaine as the hydrogen bond acceptor (HBA) and one of four polyols as the hydrogen bond donor (HBD) at different molar ratios, measured at 25 °C. 2

Table S1.4. Mole fraction solubility of p-coumaric acid (COU) in aqueous mixtures of selected deep eutectic solvents at different molar compositions and various temperatures. 3

Table S1.5. Mole fraction solubility of caffeic acid (CAF) in aqueous mixtures of selected deep eutectic solvents at different molar compositions and various temperatures. 3

Table S1.6. Mole fraction solubility of syryngic acid (SYR) in aqueous mixtures of selected deep eutectic solvents at different molar compositions and various temperatures. 4

S2. Machine learning documentation 5

Table S2.1. Description of the descriptors collected in the "SM_dataset.xlsx" file. 5

Table S2.2. Reference of the source of the solubility data. 6

S3. Detailed trajectory of the optimal model envelope and model characteristics 7

Table S3.1. Visual summary of the optimal model performance at each stage of feature pruning. Each row corresponds to a point on the optimal performance envelope (the "red line" in Figure 1 of the main text) and displays the results for the best-performing model found at that specific number of descriptors (left column). The multi-objective Pareto plot from the run that produced the best model for that complexity level. The chosen point on the envelope is highlighted. The corresponding parity plot, showing the predictive performance of that specific model on the training (black circles) and test (green circles) sets. Descriptors arranged according to decreasing importance's as determined for tentative best mode. 7

Table S3.2. The collection of parameters of final three the best models collected via DOO-IT procedure. 13

S1. Solubility measurements

Table S1.1. Mole fraction solubility of coumaric acid (COU) in deep eutectic solvents composed of choline chloride or betaine as the hydrogen bond acceptor (HBA) and one of four polyols as the hydrogen bond donor (HBD) at different molar ratios, measured at 25 °C.

	**HBA:HBD molar ratio**		
**DES**	**1:1**	**1:2**	**1:4**
ChCl - DEG	0.0644±0.0004	0.0775±0.0005	0.0706±0.0005
ChCl - TEG	0.0709±0.0006	0.0997±0.0009	0.0879±0.0006
ChCl - GLY	0.0525±0.0004	0.0642±0.0005	0.0595±0.0004
ChCl - ETG	0.0387±0.0004	0.0470±0.0003	0.0411±0.0004
Bet - DEG	0.0490±0.0005	0.0639±0.0004	0.0575±0.0005
Bet - TEG	0.0648±0.0006	0.0894±0.0005	0.0778±0.0005
Bet - GLY	0.0670±0.0005	0.0898±0.0003	0.0739±0.0004
Bet - ETG	0.0323±0.0004	0.0351±0.0002	0.0342±0.0004

Table S1.2. Mole fraction solubility of caffeic acid (CAF) in deep eutectic solvents composed of choline chloride or betaine as the hydrogen bond acceptor (HBA) and one of four polyols as the hydrogen bond donor (HBD) at different molar ratios, measured at 25 °C.

	**HBA:HBD molar ratio**		
**DES**	**1:1**	**1:2**	**1:4**
ChCl - DEG	0.0584±0.0003	0.0664±0.0005	0.0648±0.0005
ChCl - TEG	0.0740±0.0005	0.0835±0.0010	0.0824±0.0003
ChCl - GLY	0.0747±0.0004	0.0815±0.0006	0.0756±0.0005
ChCl - ETG	0.0435±0.0004	0.0494±0.0003	0.0471±0.0003
Bet - DEG	0.0497±0.0004	0.0576±0.0005	0.0537±0.0004
Bet - TEG	0.0655±0.0012	0.0755±0.0010	0.0716±0.0007
Bet - GLY	0.0561±0.0004	0.0622±0.0006	0.0573±0.0002
Bet - ETG	0.0411±0.0004	0.0458±0.0004	0.0435±0.0002

Table S1.3. Mole fraction solubility of syryngic acid (SYR) in deep eutectic solvents composed of choline chloride or betaine as the hydrogen bond acceptor (HBA) and one of four polyols as the hydrogen bond donor (HBD) at different molar ratios, measured at 25 °C.

	**HBA:HBD molar ratio**		
**DES**	**1:1**	**1:2**	**1:4**
ChCl - DEG	0.0230±0.0004	0.0465±0.0004	0.0338±0.0004
ChCl - TEG	0.0346±0.0004	0.0515±0.0004	0.0412±0.0005
ChCl - GLY	0.0213±0.0002	0.0400±0.0002	0.0310±0.0002
ChCl - ETG	0.0186±0.0002	0.0304±0.0002	0.0244±0.0002
Bet - DEG	0.0235±0.0003	0.0311±0.0002	0.0283±0.0003
Bet - TEG	0.0317±0.0003	0.0474±0.0003	0.0388±0.0003
Bet - GLY	0.0203±0.0002	0.0358±0.0003	0.0300±0.0002
Bet - ETG	0.0132±0.0001	0.0274±0.0002	0.0205±0.0002

Table S1.4. Mole fraction solubility of p-coumaric acid (COU) in aqueous mixtures of selected deep eutectic solvents at different molar compositions and various temperatures.

	ChCl – TEG 1:2			
x^*_{DES}	25°C	30°C	35°C	40°C
0.1	0.0131±0.0002	0.0168±0.0002	0.0216±0.0002	0.0287±0.0003
0.2	0.0283±0.0002	0.0357±0.0002	0.0459±0.0004	0.0604±0.0004
0.3	0.0412±0.0004	0.0506±0.0006	0.0649±0.0005	0.0862±0.0009
0.4	0.0512±0.0003	0.0664±0.0007	0.0861±0.0005	0.1133±0.0008
0.5	0.0632±0.0004	0.0790±0.0006	0.1033±0.0010	0.1413±0.0012
0.6	0.0774±0.0005	0.0946±0.0004	0.1258±0.0012	0.1692±0.0013
0.7	0.0885±0.0008	0.1119±0.0007	0.1523±0.0007	0.2060±0.0014
0.8	0.1016±0.0009	0.1348±0.0012	0.1803±0.0006	0.2401±0.0023
0.9	0.1054±0.0008	0.1368±0.0008	0.1857±0.0007	0.2507±0.0018
1.0	0.0997±0.0009	0.1304±0.0012	0.1773±0.0014	0.2433±0.0022
	Bet – GLY 1:2			
x^*_{DES}	25°C	30°C	35°C	40°C
0.1	0.0093±0.0001	0.0116±0.0001	0.0146±0.0001	0.0188±0.0001
0.2	0.0212±0.0003	0.0261±0.0002	0.0328±0.0003	0.0429±0.0003
0.3	0.0316±0.0004	0.0385±0.0003	0.0489±0.0004	0.0631±0.0003
0.4	0.0424±0.0005	0.0510±0.0004	0.0654±0.0004	0.0850±0.0007
0.5	0.0545±0.0003	0.0655±0.0004	0.0843±0.0006	0.1107±0.0012
0.6	0.0687±0.0004	0.0827±0.0006	0.1045±0.0009	0.1410±0.0021
0.7	0.0803±0.0006	0.0972±0.0008	0.1233±0.0020	0.1617±0.0014
0.8	0.0900±0.0007	0.1117±0.0011	0.1413±0.0023	0.1818±0.0015
0.9	0.0926±0.0006	0.1149±0.0011	0.1464±0.0025	0.1934±0.0021
1.0	0.0898±0.0003	0.1090±0.0010	0.1389±0.0009	0.1842±0.0017

Table S1.5. Mole fraction solubility of caffeic acid (CAF) in aqueous mixtures of selected deep eutectic solvents at different molar compositions and various temperatures.

	ChCl – TEG 1:2			
x^*_{DES}	25°C	30°C	35°C	40°C
0.1	0.0098±0.0001	0.0125±0.0001	0.0162±0.0001	0.0211±0.0001
0.2	0.0184±0.0002	0.0233±0.0002	0.0321±0.0001	0.0432±0.0002
0.3	0.0292±0.0002	0.0366±0.0003	0.0492±0.0002	0.0665±0.0004
0.4	0.0381±0.0003	0.0481±0.0003	0.0652±0.0003	0.0893±0.0002
0.5	0.0491±0.0004	0.0612±0.0003	0.0841±0.0005	0.1208±0.0009
0.6	0.0593±0.0005	0.0754±0.0003	0.1040±0.0006	0.1464±0.0014
0.7	0.0715±0.0007	0.0924±0.0007	0.1254±0.0014	0.1718±0.0012
0.8	0.0827±0.0009	0.1080±0.0013	0.1418±0.0015	0.1910±0.0018
0.9	0.0851±0.0005	0.1116±0.0017	0.1479±0.0013	0.1964±0.0019
1.0	0.0835±0.0010	0.1032±0.0014	0.1364±0.0007	0.1928±0.0017
	Bet – TEG 1:2			
x^*_{DES}	25°C	30°C	35°C	40°C
0.1	0.0077±0.0001	0.0087±0.0001	0.0101±0.0001	0.0121±0.0001
0.2	0.0155±0.0001	0.0195±0.0002	0.0203±0.0002	0.0243±0.0003
0.3	0.0243±0.0002	0.0271±0.0002	0.0318±0.0002	0.0377±0.0003
0.4	0.0343±0.0002	0.0379±0.0001	0.0450±0.0002	0.0538±0.0003
0.5	0.0432±0.0002	0.0476±0.0003	0.0565±0.0003	0.0690±0.0009
0.6	0.0543±0.0003	0.0601±0.0005	0.0703±0.0005	0.0870±0.0009

0.7	0.0659±0.0007	0.0737±0.0006	0.0862±0.0007	0.1046±0.0008
0.8	0.0797±0.0006	0.0901±0.0008	0.1042±0.0013	0.1253±0.0010
0.9	0.0823±0.0013	0.0941±0.0005	0.1123±0.0012	0.1364±0.0011
1.0	0.0755±0.0010	0.0844±0.0011	0.0989±0.0012	0.1215±0.0012

Table S1.6. Mole fraction solubility of syryngic acid (SYR) in aqueous mixtures of selected deep eutectic solvents at different molar compositions and various temperatures.

	**ChCl – TEG 1:2**			
x^*_{DES}	**25°C**	**30°C**	**35°C**	**40°C**
0.1	0.0069±0.0001	0.0093±0.0001	0.0131±0.0001	0.0174±0.0002
0.2	0.0122±0.0001	0.0158±0.0001	0.0218±0.0001	0.0305±0.0002
0.3	0.0186±0.0001	0.0249±0.0001	0.0347±0.0003	0.0476±0.0003
0.4	0.0254±0.0003	0.0347±0.0004	0.0466±0.0004	0.0643±0.0006
0.5	0.0324±0.0002	0.0446±0.0004	0.0618±0.0006	0.0822±0.0010
0.6	0.0401±0.0004	0.0524±0.0005	0.0731±0.0007	0.0992±0.0012
0.7	0.0475±0.0003	0.0628±0.0004	0.0871±0.0005	0.1214±0.0012
0.8	0.0541±0.0003	0.0699±0.0006	0.1009±0.0006	0.1351±0.0013
0.9	0.0550±0.0005	0.0718±0.0005	0.1018±0.0008	0.1373±0.0011
1.0	0.0515±0.0004	0.0660±0.0003	0.0917±0.0006	0.1234±0.0013
	**Bet – TEG 1:2**			
x^*_{DES}	**25°C**	**30°C**	**35°C**	**40°C**
0.1	0.0042±0.0001	0.0046±0.0001	0.0057±0.0001	0.0067±0.0001
0.2	0.0093±0.0001	0.0105±0.0001	0.0127±0.0001	0.0148±0.0002
0.3	0.0153±0.0001	0.0173±0.0001	0.0207±0.0002	0.0239±0.0002
0.4	0.0214±0.0002	0.0247±0.0001	0.0290±0.0002	0.0341±0.0003
0.5	0.0284±0.0002	0.0318±0.0002	0.0386±0.0002	0.0457±0.0003
0.6	0.0351±0.0003	0.0396±0.0003	0.0475±0.0009	0.0568±0.0006
0.7	0.0425±0.0003	0.0483±0.0005	0.0579±0.0010	0.0702±0.0004
0.8	0.0500±0.0002	0.0558±0.0006	0.0673±0.0015	0.0790±0.0009
0.9	0.0488±0.0004	0.0551±0.0005	0.0682±0.0014	0.0796±0.0008
1.0	0.0474±0.0003	0.0541±0.0004	0.0657±0.0011	0.0778±0.0009

S2. Machine learning documentation

The whole dataset used for machine learning purposes was collected in a spreadsheet file named SM_dataset.xlsx, which provides all necessary data for reproduction. The notation of descriptors is explained in Table S2.1

Table S2.1. Description of the descriptors collected in the "SM_dataset.xlsx" file.

descriptor	description
log(x_exp)	Experimental solubility collected as decadal logarithm of mole fraction
log(x_solub)	COSMO-RS derived solubility collected as decadal logarithm of mole fraction
ΔGfus[kJ/mol]	Values the Gibbs free energies computed fo every solute based on the fusion data: $\Delta G_{fus}=\Delta H_{fus}-T\Delta S_{fus}$ $\Delta C_{p,fus}\approx\Delta S_{fus}\approx\Delta H_{fus}/T_m$
dmu	Relative value of chemical potentials (μ): $$dmu = mu_{API} - mu_{DES}$$
dE_tot	Relative value of the total interaction energies (denoted in the output of COSMO-RS computations as "Total mean interaction energy in the mix (H_int)"): $$dE\_tot = E_{API}^{tot} - E_{DES}^{tot}$$
dE_Misfit	Relative value of the electrostatic contribution to intermolecular interaction energies (denoted in the output of COSMO-RS computations as "Misfit interaction energy in the mix (H_MF)"): $$dE\_\mathrm{Misfit} = E_{API}^{\mathrm{Misfit}} - E_{DES}^{\mathrm{Misfit}}$$
dE_HB	Relative value of the hydrogen bonding contribution to intermolecular interaction energies (denoted in the output of COSMO-RS computations as "H-Bond interaction energy in the mix (H_HB)": $$dE\_\mathrm{HB} = E_{API}^{\mathrm{HB}} - E_{DES}^{\mathrm{HB}}$$
dE_vdW	Relative value of the non-bonding contribution to intermolecular interaction energies (denoted in the output of COSMO-RS computations as "VdW interaction energy in the mix (H_vdW)": $$dE\_\mathrm{HB} = E_{API}^{\mathrm{vdW}} - E_{DES}^{\mathrm{vdW}}$$
mu1_sat	Values of chemical potential of the solutes: $$mu1\_sat = mu_{API}$$
E1_tot_sat	The values of the total interaction energies of solute $$E1\_tot\_sat = E_{API}^{tot}$$
E1_Misfit_sat	Value of the electrostatic contribution to intermolecular interaction energies the solutes: $$E1\_\mathrm{Misfit\_sat} = E_{API}^{\mathrm{Misfit}}$$
E1_HB_sat	Values of the hydrogen bonding contribution to intermolecular interaction energies of the solutes $$E1\_\mathrm{HB\_sat} = E_{API}^{\mathrm{HB}}$$
E1_vdW_sat	value of the non-bonding contribution to intermolecular interaction energies of the solutes $$E1\_\mathrm{HB\_sat} = E_{API}^{\mathrm{vdW}}$$
mu_solvent	Values of chemical potential of solvent: $$mu\_solvent = mu_{DES} = \sum_{i=1}^{N=3} x_i^* \cdot mu_i \quad \cdot$$ computed as a weighted sum of components contributions, where x_i^* represents the mole fraction of i-th component in solute free solution.

E_tot_solvent	Values of the total interaction energies of DES $$E\_tot\_solvent = E_{DES}^{tot} = \sum_{i=1}^{N=3} x_i^* \cdot E_i^{tot} \cdot$$
E_Misfit_solvent	Values of the electrostatic interaction energies of DES $$E\_tot\_solvent = E_{DES}^{Misfit} = \sum_{i=1}^{N=3} x_i^* \cdot E_i^{Misfit} \cdot$$
E_HB_solvent	Values of the hydrogen bonding interaction energies of DES $$E\_tot\_solvent = E_{DES}^{HB} = \sum_{i=1}^{N=3} x_i^* \cdot E_i^{HB} \cdot$$
E_vdW_solvent	Values of non-bonding interaction energies of DES $$E\_tot\_solvent = E_{DES}^{vdW} = \sum_{i=1}^{N=3} x_i^* \cdot E_i^{vdW} \cdot$$

Table S2.2. Reference of the source of the solubility data.

code	year	first author	DOI
1	2016	Ji	10.1016j.molliq.2016.10.110
2	2012	Manic	10.1016j.jct.2011.12.005
3	2013	Alevizou	10.1016j.jct.2013.02.013
4	2008	Mota	10.1021ie071452o
5	2016	Bitencourt	10.1016j.jct.2016.08.025
6	2020	Vilas-Boas	10.1016j.fluid.2020.112747
7	2017	Shakeel	10.1016j.molliq.2017.04.014
8	2017	Haq	10.1111jphp.12786
9	2017	Noubigh	10.1021acs.jced.7b00333
10	2019	Vilas-Boas	10.1016j.molliq.2019.111089
11	2008	Noubigh	10.1021je800205e
12	2009	Queimada	10.1021jp808683y
13	2016	Ji	10.1021/acs.jced.6b00361
14	2024	Jeliński	10.3390/molecules29163841
15	this work		
16	2024	Prinos	10.1016j.jct.2024.107335
17	2022	Prinos	10.1016/j.fluid.2022.113462

S3. Detailed trajectory of the optimal model envelope and model characteristics

This section provides a detailed, step-by-step visualization of the models that constitute the optimal performance envelope (the "red line") in Figure 1 of the main text. For each level of model complexity (i.e., for each number of descriptors from 16 down to 6), we present the multi-objective Pareto plot and the corresponding parity plot for the single best-performing model found across all eight independent DOO-IT runs at that complexity level.

This collection of figures allows for a granular inspection of the trade-off between model complexity and predictive performance. It visually documents how the model's behavior changes as features are systematically pruned, providing a complete and transparent audit trail of the evidence that led to the selection of the final 10-descriptor model.

Table S3.1. Visual summary of the optimal model performance at each stage of feature pruning. Each row corresponds to a point on the optimal performance envelope (the "red line" in Figure 1 of the main text) and displays the results for the best-performing model found at that specific number of descriptors (left column). The multi-objective Pareto plot from the run that produced the best model for that complexity level. The chosen point on the envelope is highlighted. The corresponding parity plot, showing the predictive performance of that specific model on the training (black circles) and test (green circles) sets. Descriptors arranged according to decreasing importance's as determined for tentative best mode.

Descriptors	Pareto plot	parity plots
6<br><br>log(x_solub)<br>dE_tot<br>dE_vdW<br>E1_tot_sat<br>E_Misfit_solvent<br>E_vdW_solvent	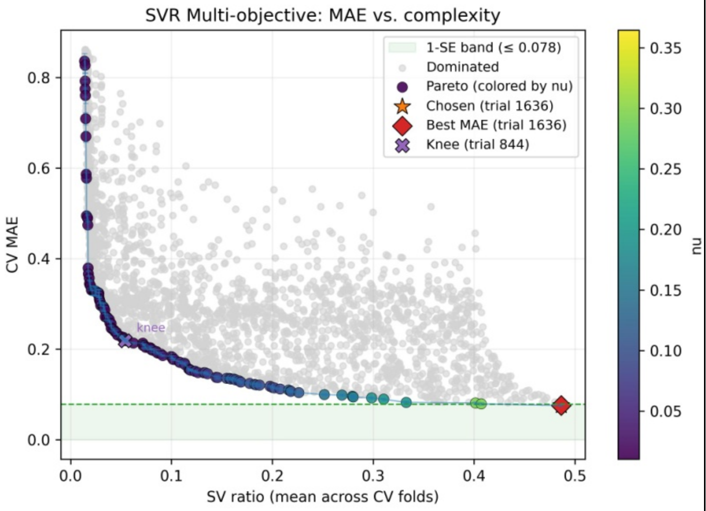	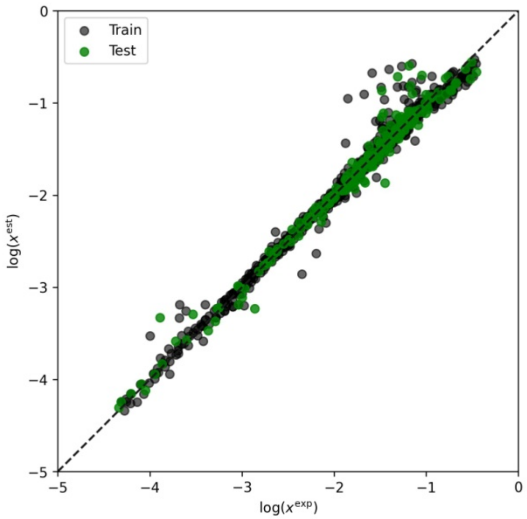
	{'nu': 0.24181105325350616, 'C': 53.18854243563865, 'log10_gamma_scale': 0.9910096384461724}	

<table>
<tr><td rowspan="2">7

E1_HB_sat
log(x_solub)
dE_tot
dE_vdW
E1_tot_sat
E_vdW_solvent
E_Misfit_solvent</td><td>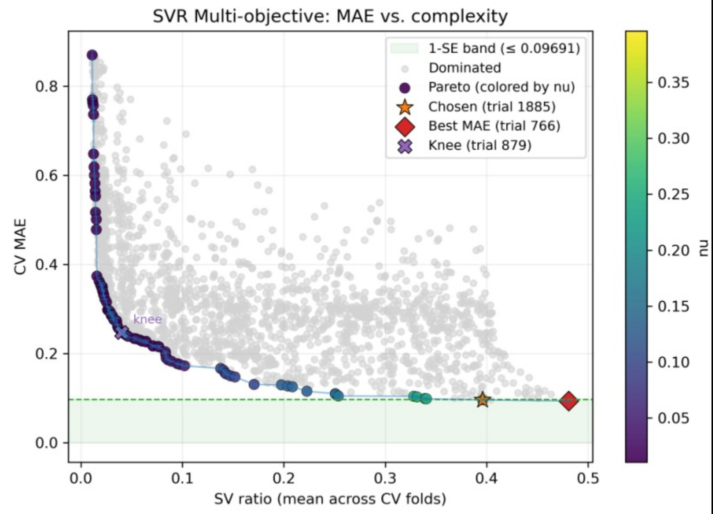
</td><td>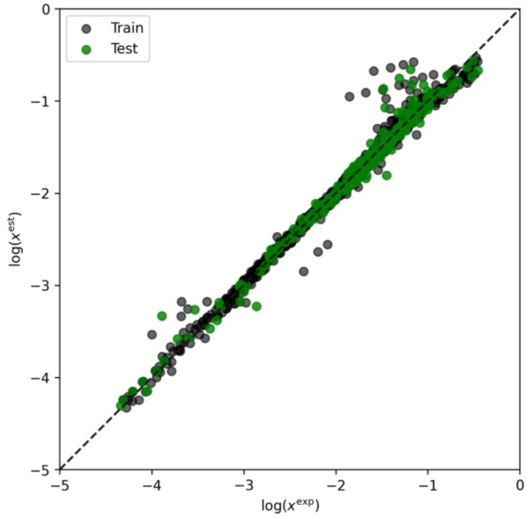
</td></tr>
<tr><td colspan="2">{'nu': 0.28132331, 'C': 50.5936558, 'log10_gamma_scale': 0.996759769}</td></tr>
<tr><td rowspan="2">8

log(x_solub)
dE_tot
dE_vdW
E1_tot_sat
E1_Misfit_sat
E1_HB_sat
E_Misfit_solvent
E_vdW_solvent</td><td>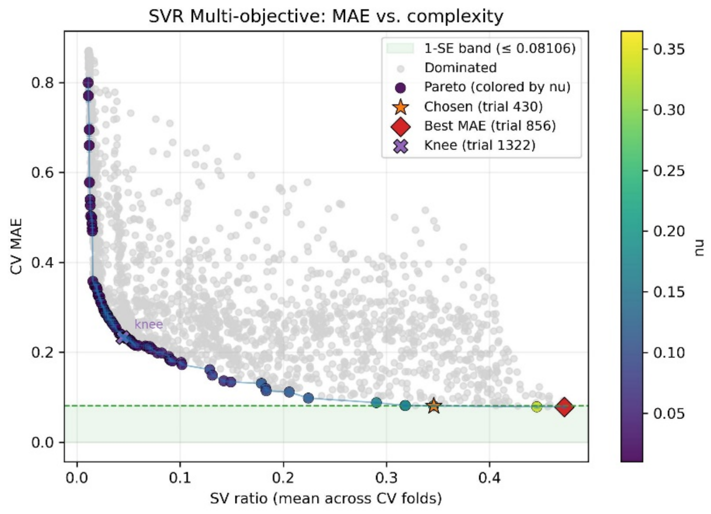
</td><td>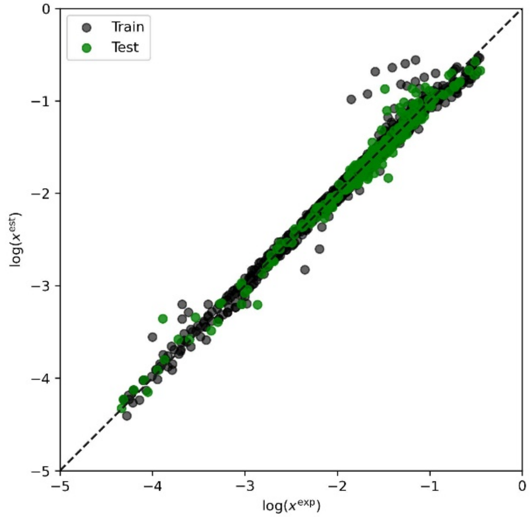
</td></tr>
<tr><td colspan="2">{'nu': 0.21614103933601256, 'C': 60.10435433984269, 'log10_gamma_scale': 0.9943767657374591}</td></tr>
</table>

<table>
<tr>
<td rowspan="2">9

log(x_solub)
dmu
dE_tot
dE_vdW
E1_tot_sat
E1_Misfit_sat
E1_HB_sat
E_Misfit_solvent
E_vdW_solvent</td>
<td>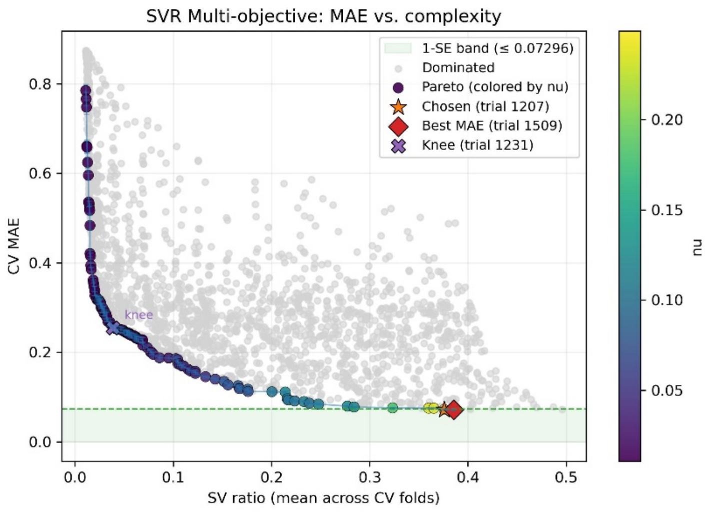
</td>
<td>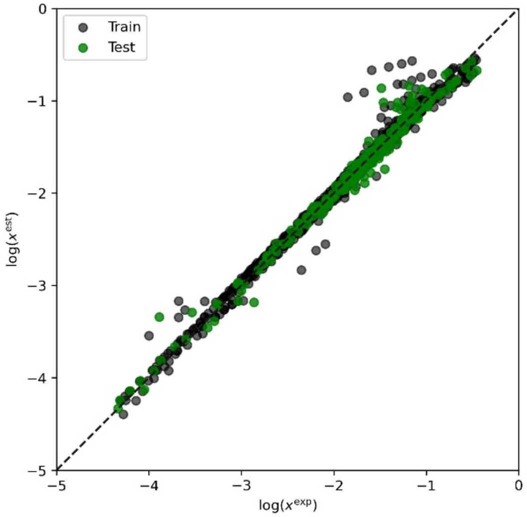
</td>
</tr>
<tr>
<td colspan="2">{'nu': 0.21688966574385077, 'C': 68.41649746223979, 'log10_gamma_scale': 0.9807157307567378}</td>
</tr>
<tr>
<td rowspan="2">10

log(x_solub)
dmu
dE_tot
dE_Misfit
dE_vdW
E1_tot_sat
E1_Misfit_sat
E1_HB_sat
E_Misfit_solvent
E_vdW_solvent</td>
<td>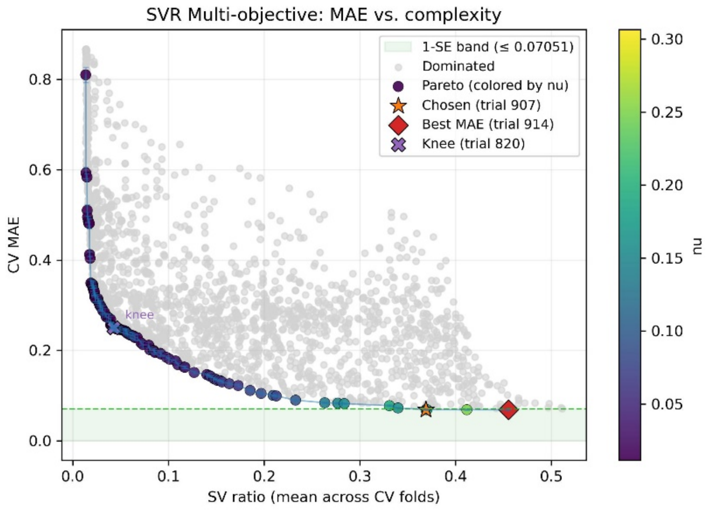
</td>
<td>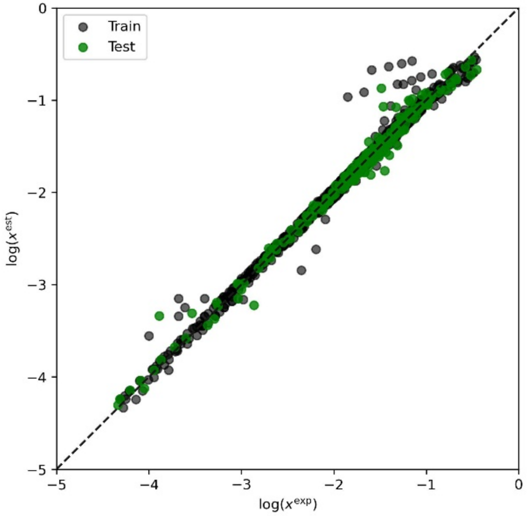
</td>
</tr>
<tr>
<td colspan="2">{'nu': 0.19386714034958083, 'C': 75.35128471154695, 'log10_gamma_scale': 0.9876318156177728}</td>
</tr>
</table>

<table>
<tr>
<td rowspan="2">11

dmu
E1_Misfit_sat
dE_Misfit
mu1_sat
E1_tot_sat
E1_HB_sat
dE_tot
log(x_solub)
E_vdW_solvent
dE_vdW
E_Misfit_solvent</td>
<td>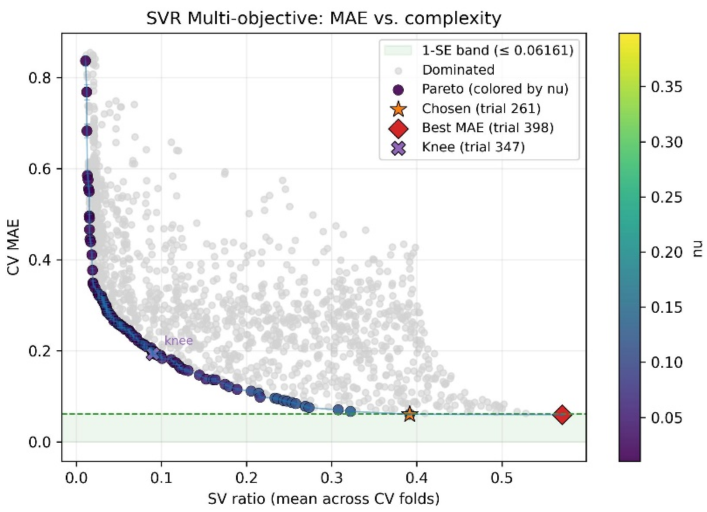
</td>
<td>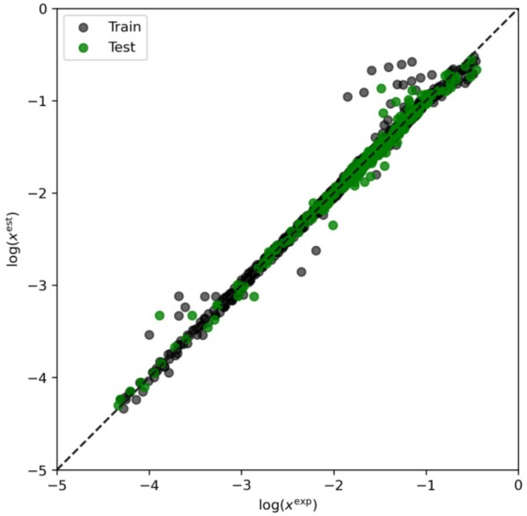
</td>
</tr>
<tr>
<td colspan="2">{'nu': 0.187771635, 'C': 69.70987474, 'log10_gamma_scale': 0.973770785}</td>
</tr>
<tr>
<td rowspan="2">12

log(x_solub)
dmu
dE_tot
dE_Misfit
dE_vdW
mu1_sat
E1_tot_sat
E1_Misfit_sat
E1_HB_sat
E_Misfit_solvent
E_HB_solvent
E_vdW_solvent</td>
<td>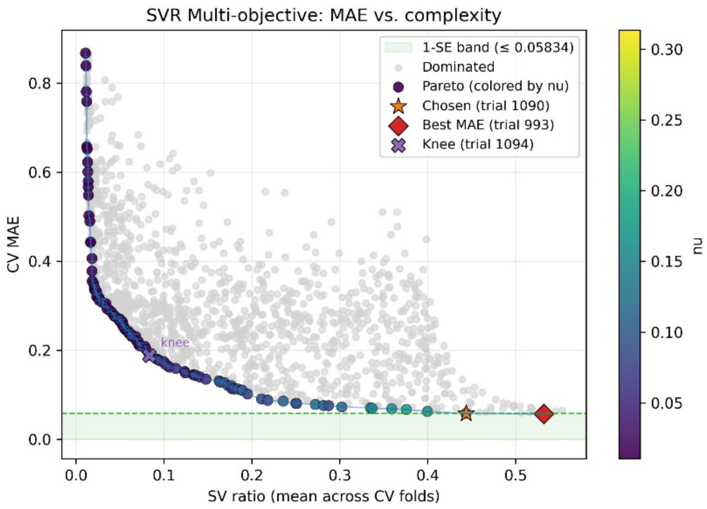
</td>
<td>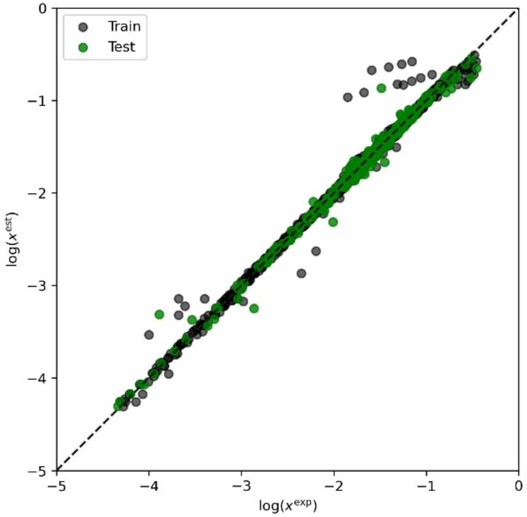
</td>
</tr>
<tr>
<td colspan="2">{'nu': 0.1944730989502946, 'C': 55.008176245073706, 'log10_gamma_scale': 0.9596251932913522}</td>
</tr>
</table>

<table>
<tr>
<td rowspan="2">13

log(x_solub)
dmu
dE_tot
dE_Misfit
dE_vdW
mu1_sat
E1_tot_sat
E1_Misfit_sat
E1_HB_sat
E1_vdW_sat
E_Misfit_solvent
E_HB_solvent
E_vdW_solvent</td>
<td>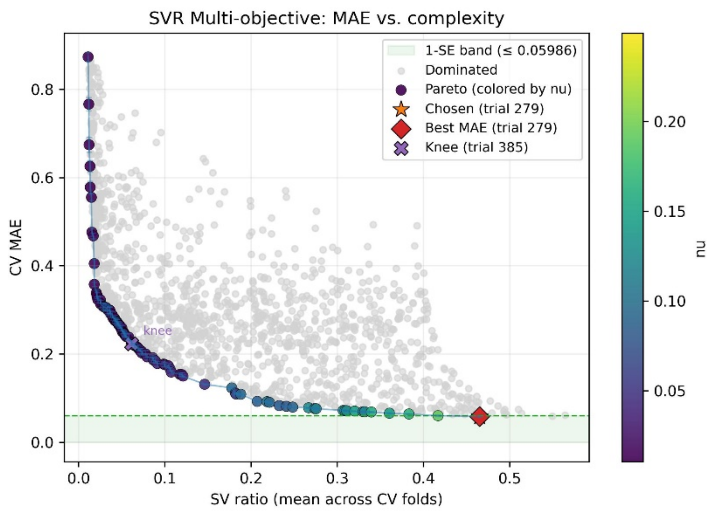
</td>
<td>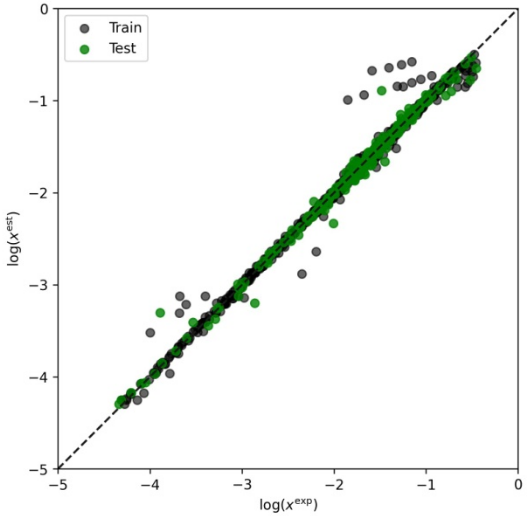
</td>
</tr>
<tr>
<td colspan="2">{'nu': 0.3389112077161621, 'C': 29.247568292653977, 'log10_gamma_scale': 0.9785912217820312}</td>
</tr>
<tr>
<td rowspan="2">14

log(x_solub)
dmu
dE_tot
dE_Misfit
dE_vdW
mu1_sat
E1_tot_sat
E1_Misfit_sat
E1_HB_sat
E1_vdW_sat
mu_solvent
E_Misfit_solvent
E_HB_solvent
E_vdW_solvent</td>
<td>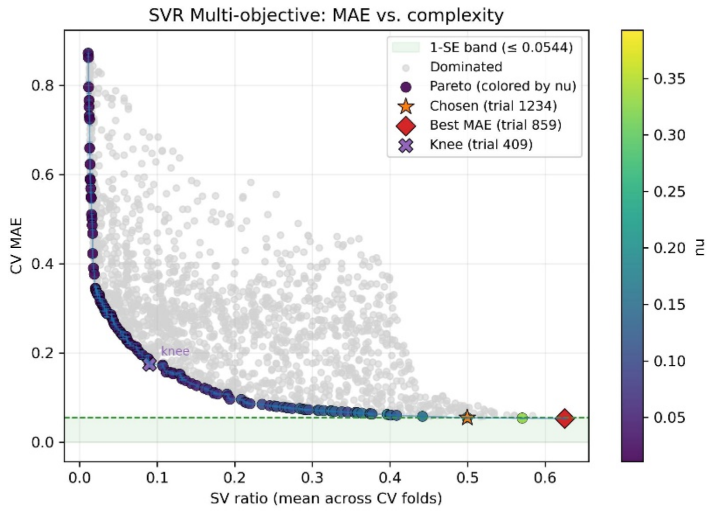
</td>
<td>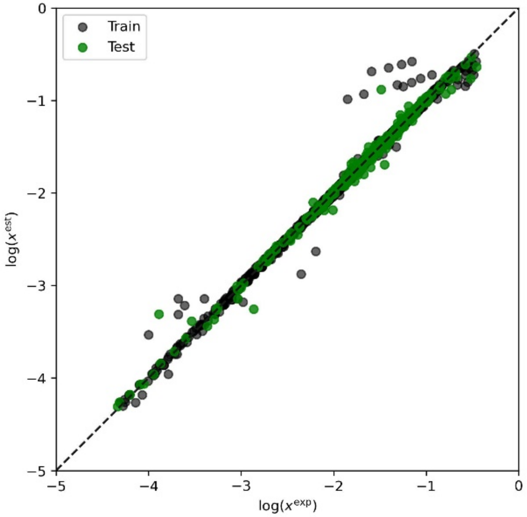
</td>
</tr>
<tr>
<td colspan="2">{'nu': 0.22939106603981135, 'C': 51.788057618457444, 'log10_gamma_scale': 0.9592679437819824}</td>
</tr>
</table>

<table>
<tr>
<td rowspan="2">15

log(x_solub)
dmu
dE_tot
dE_Misfit
dE_vdW
mu1_sat
E1_tot_sat
E1_Misfit_sat
E1_HB_sat
E1_vdW_sat
mu_solvent
E_tot_solvent
E_Misfit_solvent
E_HB_solvent
E_vdW_solvent</td>
<td>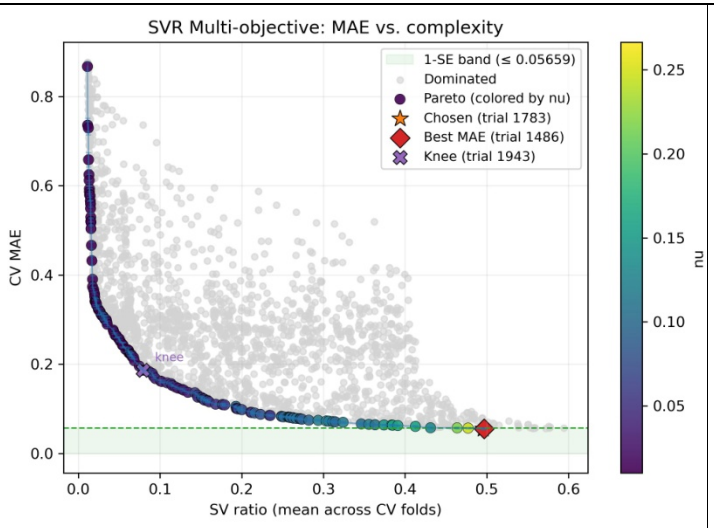</td>
<td>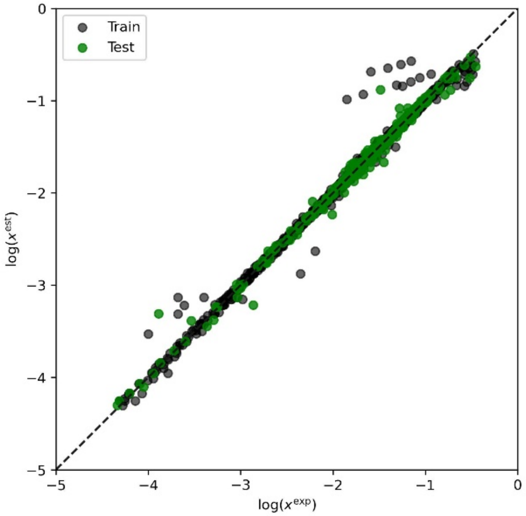</td>
</tr>
<tr>
<td colspan="2">{'nu': 0.26613945870237843, 'C': 43.36895405496951, 'log10_gamma_scale': 0.897755680308769}</td>
</tr>
<tr>
<td rowspan="2">16
log(x_solub)
dmu
dE_tot
dE_Misfit
dE_HB
dE_vdW
mu1_sat
E1_tot_sat
E1_Misfit_sat
E1_HB_sat
E1_vdW_sat
mu_solvent
E_tot_solvent
E_Misfit_solvent
E_HB_solvent
E_vdW_solvent</td>
<td>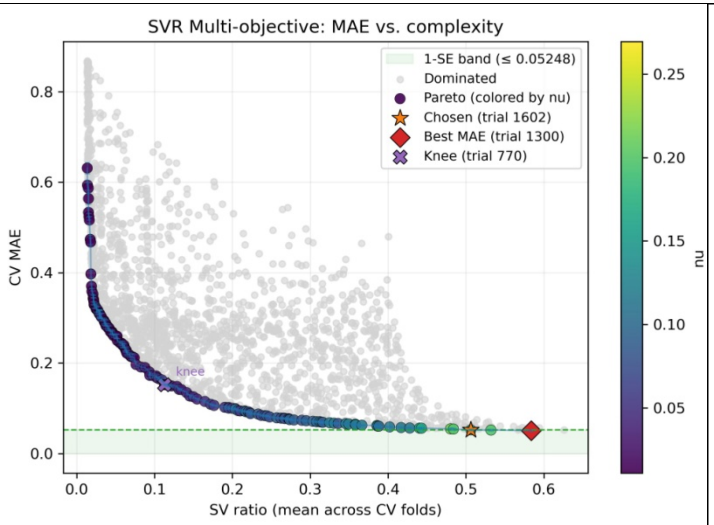</td>
<td>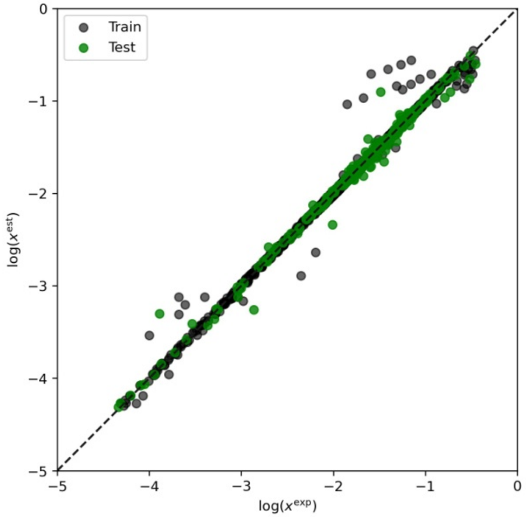</td>
</tr>
<tr>
<td colspan="2">{'nu': 0.17471449325015984, 'C': 80.53570733012884, 'log10_gamma_scale': 0.9780172112479599}</td>
</tr>
</table>

Table S3.2. The collection of parameters of final three the best models collected via DOO-IT procedure.

N_{descr}	**10**	**10**	**11**
nu	0.193867	0.19411191166	0.187772
C	75.35128	38.28563874	69.70987
log10_gamma	0.987632	0.970004	0.973771
AICc	-555.4	-530.8	-543.4
k	273	261	290
MAE±SE	0.0688±0.00116	0.0717 ± 0.001829	0.06054±0.001314
sv ratio	0.369	0.353	0.392
